# Atrial Fibrillation Prevalence Rates and Its Association with Cardiovascular–Kidney–Metabolic Factors: SIMETAP-AF Study

**DOI:** 10.3390/medicina60081309

**Published:** 2024-08-13

**Authors:** Antonio Ruiz-García, Adalberto Serrano-Cumplido, Carlos Escobar-Cervantes, Ezequiel Arranz-Martínez, Vicente Pallarés-Carratalá

**Affiliations:** 1 Lipids and Cardiovascular Prevention Unit, Pinto University Health Centre, 28320 Madrid, Spain; antoniodoctor@gmail.com; 2 Department of Medicine, European University of Madrid, 28005 Madrid, Spain; 3 Repelega Health Centre, 48920 Bizkaia, Spain; adal1953@hotmail.com; 4 Department of Cardiology, La Paz University Hospital, 28046 Madrid, Spain; 5 San Blas Health Centre, 28981 Madrid, Spain; ezequielarranz@gmail.com; 6 Department of Medicine, Jaume I University, 12006 Castellon, Spain; pallares.vic@gmail.com

**Keywords:** adults, atrial fibrillation, chronic kidney disease, heart failure, hypertension, prevalence, risk factors, stroke

## Abstract

*Background and Objectives:* Atrial fibrillation (AF) is the most frequent arrhythmia and the main cause of hospital admissions for cardioembolic stroke. The SIMETAP research project aims to update the prevalence rates of cardiovascular, renal, or metabolic factors and to evaluate their respective associations with factors that could be related. The present study aims to assess the AF prevalence rates in an adult population and its association with cardiovascular–kidney–metabolic (CKM) factors. *Materials and Methods:* This cross-sectional observational study was conducted in a primary care setting, with a population-based random sample of 6588 people aged 18.0–102.8 years. Crude and adjusted prevalence rates of AF were calculated. The associations of CKM factors with AF were assessed using bivariate and multivariate analysis. *Results:* The age- and sex-adjusted prevalence rates of AF were 2.9% in the overall adult population, 6.1% in the population aged ≥50 years, and 12.9% in the population aged ≥70 years, with no significant differences by sex. AF prevalence in the population under 50 years of age barely reached 1‰. Heart failure (HF), hypertension, chronic kidney disease (CKD), stroke, low HDL-cholesterol, and prediabetes were independent CKM factors associated with AF in the overall population, as were the same factors, except prediabetes, in the population ≥50 years old (*p* < 0.001). High or very high vascular risk was present in 92.4% [95% CI: 89.1–95.7]) of the population with AF. *Conclusions:* The adjusted prevalence rate of AF in the population aged 50 years or older was 6.1%, twice that of the overall adult population and half that of the population aged 70 years or older. The main independent CKM factors associated with AF were HF, stroke, CKD, hypertension, and low HDL-cholesterol.

## 1. Introduction

Atrial fibrillation (AF) is the most common type of supraventricular tachyarrhythmia and the leading cause of stroke and heart failure (HF). AF is caused by abnormal electrical activity within the left atrium and is characterised by uncoordinated atrial activation and consequently ineffective contraction [1,2]. Due to this irregular rhythm, blood flow becomes turbulent, increasing the likelihood of blood clots forming, which can eventually lead to a thromboembolic stroke.

AF prevalence rates increase with age, favoured by the population ageing [3]. In Europe, approximately 9 million people suffered from AF in 2010, and this rate is expected to reach 14 million in 2060 [4,5]. Over the last twenty years, the standardised incidence of AF has increased by 30% in England compared to the absolute incidence of 72% due to the ageing population [6]. The overall risk of developing AF after age 55 is 37.1%. The Amain known clinical factors involved in the etiopathology of AF include hypertension (HTN), obesity, smoking, and diabetes mellitus (DM).

The new so-called cardiovascular–kidney–metabolic (CKM) syndrome is a systemic disorder attributable to pathophysiological interactions between metabolic risk factors (obesity, adiposity, DM), chronic kidney disease (CKD), and cardiovascular diseases including HF and AF [7]. AF is a major health problem that increases cardioembolic stroke risk, causes numerous hospital admissions, increases mortality, and worsens the quality of life of patients [1,2].

Although avoiding stroke is a priority for AF patients, recent international guidelines have promoted a paradigm shift from thromboembolic risk prevention to a more comprehensive management [1,2]. European guidelines [1] advocate for the use of the “Atrial Fibrillation Better Care” (ABC) pathway [8] to streamline such an integrated approach for the care of AF patients. Briefly, the ABC pathway is based on “Avoiding” stroke, (i.e., oral “anticoagulation”), “Better” symptom management with patient-centred decisions on rate or rhythm control, and “Cardiovascular” and other “Comorbidities” management with the optimal medical therapy, including lifestyle changes [8].

The risk of stroke is increasing due to the progressive ageing of the general population and is favoured by the confluence and a similar increase in other vascular risk factors, traditionally called cardiovascular risk factors. The AFGen LT Registry [9] showed that clinical management adherent to the ABC pathway for integrated care in European patients with AF was associated with a lower risk for cardiovascular events, cardiovascular death, and all-cause death. Likewise, it also showed beneficial effects for the comprehensive management of the comorbidities assessed in this manuscript.

The main objectives of the SIMETAP research project [10] were to update population-based prevalence rates of vascular risk factors, cardiovascular diseases, CKD, or metabolic clinical conditions and evaluate with real-world data their respective associations with factors that could be related. Following this line of knowledge, the aims that led to carrying out this research were to update the AF prevalence rates in Spanish populations of both overall adults and those aged 50 or older and to assess whether factors related to CKM syndrome are associated with AF.

## 2. Material and Methods

SIMETAP-AF is a multicentre, cross-sectional, observational sub-study of the SIMETAP study, whose design was previously reported [10], authorised by the Health Service from the Madrid Community (Spain), and carried out by 121 physicians (Acknowledgments) from 64 primary healthcare centres. All patients of the research physicians aged 18 or older were included in a simple random sampling performed by the Excel’s randbetween function. Inclusion criteria: The order indicated by the lists of random numbers was applied to include the study subjects until reaching the sample size necessary to evaluate the objectives of this study. Patients with terminal illnesses, moderate or severe cognitive impairment, dementia, schizophrenia, moderate or severe psychosis, residing in nursing homes or social institutions for the elderly, immobilised at home who cannot go to a medical consultation, pregnant women, and people who were participating in other clinical studies or declined inclusion in this study were excluded per protocol. Finally, 6588 people aged 18 years or over were recruited with informed consent and with the necessary clinical and laboratory data to be evaluated (response rate 62.9%) (extended information in Supplementary Materials). All information assessed in this study was collected from the primary care electronic health records under real-world data settings.

The registry of AF diagnosis (ICD-10-CM code: I48; ICPC-2 code: K78) [11,12] in the electronic health records of patients was considered as AF, without differentiating by phenotypes (paroxysmal, persistent, or permanent). The concepts and criteria of the assessed clinical conditions and CKM factors are shown in Table S1.

Qualitative variables indicated the number and percentage of each category, using the Chi-square test and odds ratios (ORs) and calculating the lower and upper limits of the bilateral 95% confidence interval (CI). The Shapiro–Wilk test was used to check the data fitting to normal distribution for continuous variables. If the variables showed normal distribution, they were analysed using the arithmetic mean and standard deviation (SD) and compared using Student’s *t*-test or an analysis of variance. The median and interquartile range (IQR) of age were determined. Cohen’s d was used to assess the standardised mean difference, considering the effect size according to the proximity to the following d-values: 0.2 small; 0.5 medium; 0.8 large. The age- and sex-adjusted prevalence rates were calculated by the direct method (extended information in Supplementary Materials). To assess the individual effect of comorbidities and clinical conditions on the dependent variable (AF), multivariate logistic regression analysis was performed using the backward stepwise method, initially introducing into the model all the variables that showed association in the bivariate analysis up to a *p*-value < 0.10, except for complex variables such as metabolic syndrome (MetS) [13] and CUN-BAE (according to its acronym in Spanish, Clínica Universitaria de Navarra—Body Adiposity Estimator) adiposity [14], which include parameters that were individually assessed in the analysis, and for erectile dysfunction, because it only affects men. Subsequently, the variable that contributed the least to the fit of the analysis was eliminated at each step. Bivariate and multivariate analyses were performed for both the overall adult population and the population aged 50 or older. All tests were two-tailed, and a *p*-value < 0.05 was considered significant. Statistical analysis was performed using the SPSS for Windows, version 25 (IBM, Armonk, NY, USA).

## 3. Results

**Figure 1:**
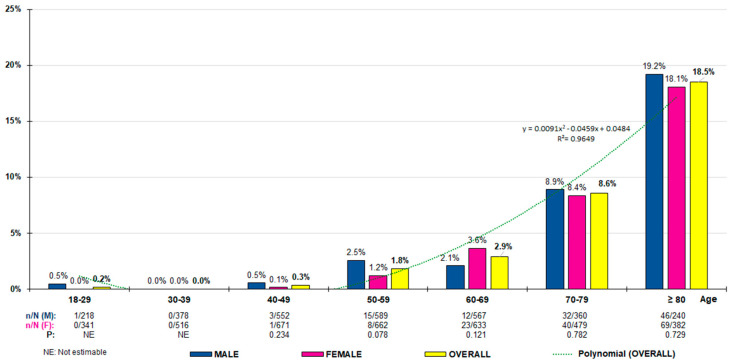
Atrial fibrillation prevalence rates according to age groups. n: number of cases; N: sample size; M: male; F: female; *p: p*-value of the difference in percentages (M–F).

The crude prevalence rates of AF were 3.8% (95% CI 3.3–4.3) in the overall adult population, with no significant (*p* = 0.866) differences between males (3.8% [95% CI 3.1–4.4]) and females (3.8% [95% CI 3.2–4.4]); 6.3% (95% CI 5.5–7.0) in people aged 50 or over, with no significant (*p* = 0.513) differences between males (6.0% [95% CI 4.9–7.1]) and females (6.5% [95% CI 5.4–7.5]); and 12.8% (95% CI 11.1–14.5) in people aged 70 or over, with no significant (*p* = 0.848) differences between males (13.0% [95% CI 10.3–15.7]) and females (12.7% [95% CI 10.4–14.9]). The age- and sex-adjusted prevalence rates of AF were 2.9% (2.7% [male] vs. 3.1% [female]) in the overall adult population, 6.1% (5.7% [male] vs. 6.4% [female]) in the population aged 50 or over, and 12.9% (12.9 [male] vs. 13.0% [female]) in the population aged 70 or over. The adjusted prevalence rate in the population under 50 years of age was 0.09% (Table S2. Supplementary Materials). The distribution of the age groups’ prevalence rates of AF increased precisely according to the polynomial function *y =* 0.0091x^2^ − 0.0459x + 0.0484 (R² = 0.965), with no significant differences between men and women (Figure 1).

The differential clinical characteristics between subjects with and without AF are shown in Table 1. The median (IQR) ages of the adult populations with and without AF were 78.8 (69.6–84.6) years and 53.9 (41.1–66.9) years, respectively, and 79.1 (70.6–84.7) years and 65.1 (57.5–74.4) years, respectively, in populations aged 50 or over. Among the AF population, the difference in the mean [SD] ages between women (78.1 [10.7] years) and men (74.7 [12.4] years) was significant (*p* = 0.022) in adults aged 18 or over but not in populations aged 50 or over (78.3 [10.4] years in females vs. 75.9 [10.9] years in males; *p* = 0.081). Most quantitative clinical variables were significantly higher in the AF populations than in the population without AF, except for total cholesterol, high-density lipoprotein cholesterol (HDL-C), non-HDL-C, low-density lipoprotein cholesterol (LDL-C), non-HDL-C/HDL-C, and estimated glomerular filtration rate, which were significantly higher both in the adult population and in the population aged 50 or over without AF, and also for diastolic blood pressure and alanine-aminotransferase in the population aged 50 or over without AF (Table 1). Most CKM factors and other variables showed a significant association with AF in both the adult population and population aged 50 or over, except for alcoholism in both populations, overweight in the adult population, and prediabetes, hypercholesterolaemia, and hypertriglyceridaemia in subjects aged 50 or over. Glycaemic-lowering drug therapy (GLT) and urate-lowering drug therapy (ULT) were used similarly in both adult populations with and without AF, and only ULT was used similarly in populations aged 50 or over with and without AF. Additionally, blood pressure-lowering drug therapy (BPLT) and lipid-lowering therapy (LLT) were used at a higher rate in the adult AF population, and GLT, BPLT, and LLT were used at a higher rate in the AF population aged 50 or over. Very high “vascular risk” (VR)—traditionally referred to as cardiovascular risk—showed a strong association with AF in both the adult population and population aged 50 or over, unlike current smoking and moderate VR, which showed a significant association in both populations without AF, and low VR, which also showed a significant association in the adult population without AF. Almost the entire population with AF had high or very high VR (92.4% [95% CI: 89.1–95.7] in adults and 93.5% [95% CI: 90.4–96.6] in those aged 50 or over) (Table 2).

**Figure 2:**
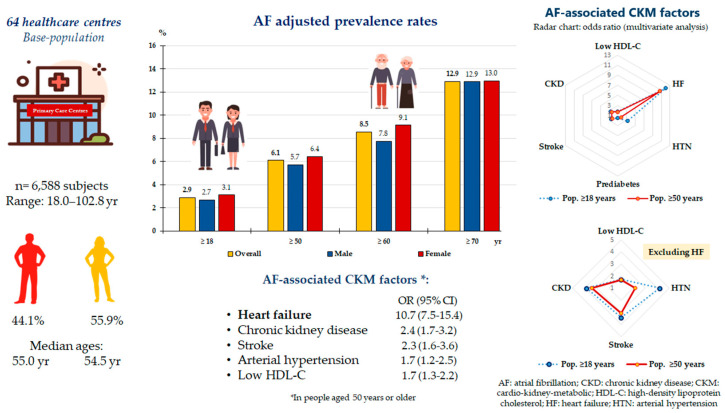
Prevalence rates of AF and its association with CKM factors.

**Figure 3:**
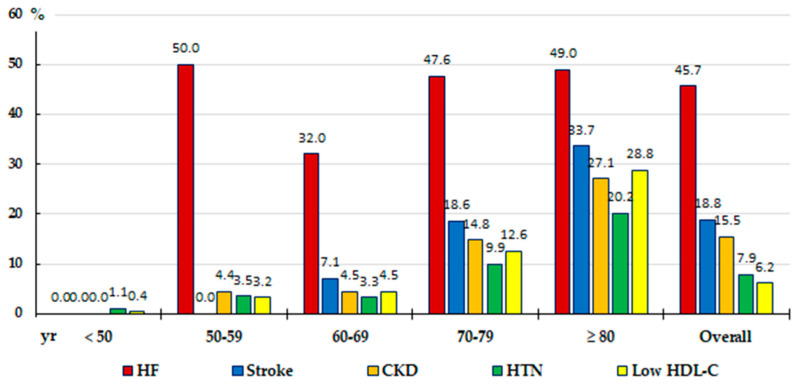
A comparison of the prevalence rates of the main AF-related CKM factors according to age groups. AF: atrial fibrillation; CKD: chronic kidney disease; CKM: cardiovascular–kidney–metabolic; HDL-C: high-density lipoprotein cholesterol; HTN: arterial hypertension.

Multivariate analysis showed that HF stood out as the most important independent factor associated with AF in both the adult population and those aged 50 or over. Other independent factors associated with AF were HTN, CKD, stroke, and low HDL-C in both populations, as well as prediabetes in the adult population (Table 3), although only HTN, CKD, stroke, and low HDL-C were significant when HF was ruled out in the multivariate analysis (Figure 2). Figure 3 shows a comparison of the prevalence rates of the main factors independently associated with AF according to age groups, which are anecdotal in the population under 50 years of age. HF stood out in all the remaining groups 50–59, 60–69, 70–79, and over 80 years of age. The prevalence rates of stroke, CKD, low HDL-C, and HTN were lower in the 50–59 and 60–69 age groups, almost triple in the 70–79 age group compared to the 60–69 age group, and double in the 80 or older age group compared to the 70–79 age group.

## 4. Discussion

Our study showed that AF prevalence increased progressively with age (Figure 1 and Figure 2), especially after the age of 50, which is similar to the results of other studies [3,4,5,6]. The worldwide prevalence of AF is increasing, ranging between 1% and 3% in the adult population [1,2,3,4,5,6]. The AF adjusted prevalence in our study ranged from 2.9% in overall adults to 12.9% in people aged 70 years or older. A study based on population-based data from 195 countries showed that the AF incidence increased progressively with age, reaching its maximum level between 75 and 79 years in both sexes, being higher in men up to 80 years of age and in women after this age [6]. In contrast, our study showed that the adjusted prevalence rates were slightly higher with non-significant differences in women than in men in the younger age groups (≥18, ≥50, ≥60 years) and were similar in the population aged 70 years or older. Our results are similar to those shown in the OFRECE study, which reported an AF prevalence rate of 4.4% in the Spanish population over 40 years of age and 17.7% in the population over 80 years of age [15].

Our study showed that the presence of CKM factors was higher in AF patients compared to those without AF and that HF, CKD, stroke, HTN, and low HDL-C were the risk factors independently associated with AF (Table 3). Smoking increases the risk of stroke [16] and is associated with an increased incidence of AF, whereas avoiding smoking decreases the risk of AF [17]. Of note, only 6.5% of AF patients aged 50 or older in our study continued to smoke, compared with 24% of stroke survivors in the United States who continued to smoke [18]. In an analysis of the ACTIVE-A, RE-LY, and AVERROES trials, AF was found to be the most common cause of new-onset HF [19]. AF is primarily associated with an increased risk of HF because both are pathologies that often coexist and predispose each other [20]. Our study highlighted that HF is the comorbidity that is most strongly and independently associated with AF and vice versa [21]. Moreover, stroke and HF can be the cause and consequence of each other [22], and their coexistence increases mortality [23]. Laupacis et al. [24] showed that the risk of stroke in AF patients varied from 1% in those under 65 years of age to 8.5% in those over 75 years of age. Our study also showed that stroke increased with age and was independently associated with AF.

As reported by other studies [25], our data showed that 81% of patients with AF had HTN. HTN alone increased the incidence of AF by 50% [26], increased the risk of developing AF by 50% [27], and could explain up to 22% of the AF prevalence [28]. Morseth et al. showed that HTN was associated with AF in the age groups older than 60 years but not in those younger than that age [29].

The European MORGAN/BiomarCaRE study reported that CKD was an independent risk factor for AF and HF [30]. The ENRICA study found a CKD prevalence of 15.5% of the Spanish adult population [31]. Our data were similar in people aged 50 years or older without AF. In a previous study [32], we reported that the AF prevalence rate among patients with CKD was 14.9%. Tomaszuk-Kazberuk et al. found a CKD prevalence rate of 26.0% among AF patients hospitalised due to coronary angiography [33]. In contrast, our CKD prevalence rate reached 46.1% among AF patients aged 50 or older.

Metabolic factors are also linked to AF-related cardiac and renal factors, expanding the pathophysiological connection between CKM syndrome and AF. A recent network meta-analysis including 108,026 patients showed that non-cardiac systemic factors play a role in AF, sodium−glucose cotransporter-2 inhibitors reduced the risk of incident AF, and the use of dapagliflozin was associated with a significant reduction in AF risk [34]. There are pathophysiological mechanisms that relate disorders of glycaemic metabolism to AF [35]. The Framingham study showed that DM was an independent risk factor for AF [36], and a recent study showed that prediabetes was also an independent risk factor for AF [37]. Although our study showed that both prediabetes and DM were more common in AF patients than in those without AF, the multivariate analysis showed that only prediabetes was associated with AF when evaluating the entire population older than 18 years, and neither DM nor prediabetes appeared as independent factors associated with AF when assessing the population aged 50 or older. Our study showed that 33% of AF patients had DM, compared with 20% found in a Japanese study [38]. The higher prevalence of DM in our population without AF aged 50 years or older (23.3%) compared to the Japanese population without AF aged 40 years or older could explain these differences. Obesity and adiposity favour both the appearance and recurrence of AF [39]. The REVERSE-AF study showed that weight loss reverses the progression of AF in overweight or obese patients, especially when this loss exceeds 10% [40]. We found a high prevalence of physical inactivity, obesity, abdominal obesity, adiposity, prediabetes, DM, HTN, low HDL-C, and hypertriglyceridaemia among AF patients; hence, it is logical that there was also a high prevalence of MetS (78.8%). A meta-analysis showed that MetS was associated with an increased risk of AF, as well as most of its components but not triglycerides [41].

In our study, LLT was used more frequently in the AF population due to the high prevalence of dyslipidaemia. This could justify that only low HDL-C was found to be associated with AF in the multivariate analysis, probably because LLT causes less variations in plasma HDL-C concentrations. Morseth et al. also found no association in the multivariate analysis between elevated cholesterol levels and the incidence of AF [29]. Some studies have shown that high concentrations of LDL-C and total cholesterol decrease the AF risk during the first five years of follow-up, although not longer, but low HDL-C and high triglyceride concentrations are associated with an increased risk of new-onset AF [42,43].

The main limitations of this study were the inability to determine causality or to estimate incidence rates, inter-interviewer variability, possible under-reporting of an AF diagnosis in electronic health records, possible heterogeneity of the measurement and laboratory equipment, and AF underdiagnoses due to the exclusion of study subjects per protocol that may have biased the results. The values of the variables that were not reported in all study subjects were few, occurred at random, and were similar in the comparison groups, although this could imply a minimal confounding factor in the analysis of the difference between patients with and without AF. Left ventricular ejection fraction could not be assessed because this variable was not recorded in the primary care electronic health records of patients with HF. The results reporting associations between many factors or clinical conditions and the presence of AF should be interpreted as speculative and with caution, because multiple comparisons using ORs and 95% CI could increase the risk of obtaining a statistically significant association by chance (familywise error or alpha-inflation phenomenon). The results from BPLT, LLT, and GLT should not be considered particularly relevant in the population aged 50 years or older, since they similarly reflect the different prevalences of risk factors and comorbidities that are treated with these specific agents. On the other hand, the differences in serum uric acid concentrations would not be biased because the ULT was similar in the populations with and without AF. The key strengths of this study include a large population-based sample, the updating of both crude and adjusted AF prevalence rates in people aged 18 to 102 years, and the assessment of the association of AF with numerous CKM factors. Since the prevalence of AF in the population under 50 years of age does not even reach 1‰, we think it is better to focus further studies on AF with populations aged 50 years or older.

Among the clinical implications of our study, it is worth highlighting that AF is strongly influenced by age; hence, its prevalence rates should be age-adjusted to compare among countries. Likewise, assessing the epidemiological magnitude of AF is essential to better plan prevention policies aimed at reducing the high healthcare and economic burden mainly due to the increase in admissions for cardioembolic stroke, optimising available health resources and improving the quality of life of the patients. Finally, because almost a third of the AF population is asymptomatic, enhancing AF detection, promoting healthy lifestyles, and controlling VR factors are essential for AF management [44,45]. We hope that the results of our study update AF prevalence and help to better understand the magnitude and importance of CKM factors associated with AF.

## 5. Conclusions

AF is a serious health problem worldwide, closely related to ageing, which clusters many comorbidities that accelerate its progression and increase the risk of mortality and hospitalisation due to stroke. Our study presents the results of a randomised cohort study of Spanish patients, broken down by age and other characteristics, as well as by CKM factors associated with the presence of AF, updating the results of previous epidemiological studies, which are increasing due to the progressive ageing of the general population and confirming the complexity of patients diagnosed with AF, who had a greater burden of comorbidities and CKM factors. The AF prevalence in the population under 50 years of age barely reaches 1‰. The adjusted prevalence rate of AF in the population aged 70 years or older is close to 13%, doubling that of the population aged 50 years or older and quadrupling that of the overall adult population, with non-significant differences according to sex. Most factors related to CKM syndrome and especially HF show a strong association with AF. Ageing and the associated CKM factors of AF patients determine that 93% of them have a high VR. The early detection of AF-related clinical conditions and comorbidities in addition to their comprehensive management according to the ABC pathway recommended by European guidelines could prevent stroke and the worsening of HF and delay the progression of CKD.

## Figures and Tables

**Table 1:** Clinical characteristics of populations with and without atrial fibrillation.

	Population ≥ 18 Years					Population ≥ 50 Years					
	With AF		Without AF		*p*	With AF		Without AF		*p*	Cohen’s *d* (95% CI)
	No.	Mean (SD)	No.	Mean (SD)		No.	Mean (SD)	No.	Mean (SD)		
Age (yr)	250	76.6 (11.5)	6338	54.3 (17.2)	<0.001	245	77.3 (10.7)	3667	66.3 (10.8)	<0.001	1.0 (0.9; 1.1)
BMI (kg/m^2^)	250	29.5 (5.4)	6338	27.4 (5.1)	<0.001	245	29.4 (5.4)	3667	28.5 (4.9)	0.006	0.2 (0.1; 0.3)
Waist circumference (cm)	250	100.2 (13.6)	6338	93.1 (14.0)	<0.001	245	100.1 (13.5)	3667	96.7 (13.0)	<0.001	0.3 (0.1; 0.4)
WHtR	250	0.62 (0.09)	6338	0.57 (0.09)	<0.001	245	0.63 (0.09)	3667	0.60 (0.08)	<0.001	0.4 (0.2; 0.5)
Adiposity CUN-BAE (%)	250	39.5 (8.0)	6338	34.6 (8.6)	<0.001	245	39.6 (7.9)	3667	37.3 (7.8)	<0.001	0.3 (0.2; 0.4)
SBP (mmHg)	250	126.2 (15.3)	6338	121.8 (15.4)	<0.001	245	126.3 (15.3)	3667	126.5 (14.7)	0.837	0.0 (0.1; −0.1)
DBP (mmHg)	250	72.5 (8.9)	6338	73.4 (9.8)	0.178	245	72.5 (8.9)	3667	75.0 (9.3)	<0.001	−0.3 (−0.4; −0.1)
FPG (mg/dL) ^a^	250	104.0 (29.3)	6338	95.7 (25.8)	<0.001	245	104.5 (29.4)	3667	101.9 (28.4)	0.175	0.1 (0.0; −0.2)
HbA_1c_ (%) ^b^	224	6.02 (0.95)	5009	5.62 (0.89)	<0.001	219	6.04 (0.95)	3060	5.85 (0.96)	0.006	0.2 (0.1; 0.3)
Total cholesterol (mg/dL) ^c^	250	177.4 (41.6)	6338	193.4 (39.1)	<0.001	245	176.8 (41.2)	3667	197.5 (38.8)	<0.001	−0.5 (−0.7; −0.4)
HDL-C (mg/dL) ^c^	250	52.0 (16.0)	6338	54.9 (14.6)	0.002	245	52.1 (16.1)	3667	55.1 (14.8)	0.002	−0.2 (−0.3; −0.1)
LDL-C (mg/dL) ^c^	247	100.2 (35.2)	6279	114.7 (34.4)	<0.001	243	99.9 (35.0)	3636	117.4 (34.4)	<0.001	−0.5 (−0.6; −0.4)
Non-HDL-C (mg/dL) ^c^	250	125.4 (38.7)	6338	138.4 (38.3)	<0.001	245	124.7 (38.1)	3667	142.5 (36.9)	<0.001	−0.5 (−0.6; −0.3)
TG (mg/dL) ^d^	250	124.9 (68.3)	6338	120.3 (83.7)	0.401	245	123.3 (65.7)	3667	127.1 (75.9)	0.451	−0.1 (−0.2; 0.1)
Non-HDL-C/HDL-C	250	2.63 (1.11)	6338	2.72 (1.13)	0.193	245	2.61 (1.10)	3667	2.78 (1.07)	0.015	−0.2 (−0.3; 0.0)
TG/HDL-C	250	2.77 (2.03)	6338	2.52 (2.57)	0.124	245	2.74 (2.00)	3667	2.64 (2.28)	0.526	0.1 (−0.1; 0.2)
SUA (mg/dL) ^e^	245	5.57 (1.69)	6244	4.94 (1.47)	<0.001	240	5.60 (1.67)	3619	5.14 (1.47)	<0.001	0.3 (0.2; 0.4)
AST (U/L)	183	22.9 (8.7)	4638	23.1 (43.9)	0.951	178	22.8 (8.4)	2674	23.7 (42.1)	0.785	−0.1 (−0.3; 0.0)
ALT (U/L)	244	23.0 (14.1)	6178	24.9 (17.0)	0.074	239	22.5 (13.1)	3568	25.0 (16.0)	0.018	−0.2 (−0.3; 0.0)
GGT (U/L)	236	45.9 (54.1)	5872	32.9 (50.6)	<0.001	231	45.5 (53.1)	3393	36.1 (44.9)	0.002	0.2 (0.1; 0.3)
Fatty liver index	236	59.6 (27.6)	5872	44.5 (30.5)	<0.001	231	59.4 (27.6)	3393	52.5 (28.4)	<0.001	0.2 (0.1; 0.4)
Creatinine (mg/dL) ^f^	250	0.96 (0.32)	6338	0.84 (0.29)	<0.001	245	0.97 (0.32)	3667	0.86 (0.32)	<0.001	0.3 (0.2; 0.5)
eGFR (mL/min/1.73 m^2^)	250	69.3 (20.3)	6338	91.4 (20.1)	<0.001	245	68.5 (19.8)	3667	82.2 (17.6)	<0.001	−0.8 (−0.9; −0.6)
uACR (mg/g) ^g^	250	46.2 (124.7)	6338	15.2 (56.1)	<0.001	245	47.0 (125.8)	3667	19.1 (67.8)	<0.001	0.4 (0.3; 0.5)

SD: standard deviation; *p*: *p*-value of the difference in means. Cohen’s d: standardised mean difference (effect size according to d: 0.2 small; 0.5 medium; 0.8 large); CI: confidence interval; AF: atrial fibrillation; ALT: alanine aminotransferase; AST: aspartate aminotransferase; BMI: body mass index; CUN-BAE: according to its acronym in Spanish, Clínica Universitaria de Navarra—Body Adiposity Estimator; DBP: diastolic blood pressure; eGFR: estimated glomerular filtration rate; FPG: fasting plasma glucose; GGT: gamma-glutamyl transferase; HbA_1c_: glycated haemoglobin A_1c_; HDL-C: high-density lipoprotein cholesterol; LDL-C: low-density lipoprotein cholesterol; SBP: systolic blood pressure; SUA: serum uric acid; TG: triglyceride; uACR: urine albumin–creatinine ratio; WHtR: waist-to-height ratio. The definitions of the cardiovascular–kidney–metabolic factors, comorbidities, or clinical conditions are shown in Table S1 (Supplementary Materials). ^a^ To convert from mg/dL to mmol/L, multiply by 0.05556; ^b^ to convert from % (DCCT) to mmol/mol (IFCC), subtract 2.15 and multiply by 10.929; ^c^ to convert from mg/dL to mmol/L, multiply by 0.02586, ^d^ to convert from mg/dL to mmol/L, multiply by 0.01129; ^e^ to convert from mg/dL to mmol/L, multiply by 0.05948; ^f^ to convert from mg/dL to mmol/L, multiply by 0.08842; ^g^ to convert from mg/g to mg/mmol, multiply by 0.01131.

**Table 2:** Cardiovascular–kidney–metabolic factors in populations with and without AF.

	Population ≥ 18 Years				Population ≥ 50 Years			
	With AF No. (%) N = 250	Without AF No. (%) N = 6338	OR (95% CI)	*p*	With AF No. (%) N = 245	Without AF No. (%) N = 3667	OR (95% CI)	*p*
Male	109 (43.6)	2795 (44.1)	1.0 (0.8–1.3)	0.876	105 (42.9)	1651 (45.0)	0.9 (0.7–1.2)	0.509
Current smoking	17 (6.8)	1409 (22.2)	0.3 (0.2–0.4)	<0.001	16 (6.5)	649 (17.7)	0.3 (0.2–0.5)	<0.001
Alcoholism	21 (8.4)	589 (9.3)	0.9 (0.6–1.4)	0.633	18 (7.3)	339 (9.2)	0.8 (0.5–1.3)	0.318
Physical inactivity	145 (58.0)	2934 (46.3)	1.6 (1.2–2.1)	<0.001	141 (57.6)	1736 (47.3)	1.5 (1.2–2.0)	0.002
Overweight	90 (36.0)	2426 (38.3)	0.9 (0.7–1.2)	0.467	87 (35.5)	1573 (42.9)	0.8 (0.6–1.0)	0.024
Obesity	107 (42.8)	1726 (27.2)	2.0 (1.5–2.6)	<0.001	105 (42.9)	1245 (34.0)	1.5 (1.1–1.9)	0.005
Abdominal obesity	164 (65.6)	2758 (43.5)	2.5 (1.9–3.2)	<0.001	161 (65.7)	2002 (54.6)	1.6 (1.2–2.1)	0.001
Excess adiposity CUN-BAE	239 (95.6)	4593 (72.5)	8.3 (4.5–15.1)	<0.001	234 (95.5)	3314 (90.4)	2.3 (1.2–4.2)	0.007
High WHtR	197 (78.8)	3499 (55.2)	3.0 (2.2–4.1)	<0.001	193 (78.8)	2594 (70.7)	1.5 (1.1–2.1)	0.007
Prediabetes	80 (32.0)	1369 (21.6)	1.7 (1.3–2.2)	<0.001	77 (31.4)	1036 (28.3)	1.2 (0.9–1.5)	0.286
Diabetes	83 (33.2)	953 (15.0)	2.8 (2.1–3.7)	<0.001	82 (33.5)	854 (23.3)	1.7 (1.3–2.2)	<0.001
Hypertension	202 (80.8)	2345 (37.0)	7.2 (5.2–9.9)	<0.001	199 (81.2)	2081 (57.7)	3.3 (2.4–4.6)	<0.001
Hypercholesterolaemia	193 (77.2)	3908 (61.7)	2.1 (1.6–2.8)	<0.001	190 (77.6)	2840 (77.4)	1.0 (0.7–1.4)	0.970
Low HDL-C	113 (45.2)	1706 (26.9)	2.2 (1.7–2.9)	<0.001	110 (44.9)	1039 (28.3)	2.1 (1.6–2.7)	<0.001
Hypertriglyceridaemia	95 (38.0)	1852 (29.2)	1.5 (1.1–1.9)	0.003	92 (37.6)	1299 (35.4)	1.1 (0.8–1.4)	0.501
Hyperuricaemia ^a,b^	49 (20.0)	691 (11.1)	2.0 (1.5–2.8)	<0.001	49 (20.4)	490 (13.5)	1.6 (1.2–2.3)	0.003
Fatty liver index ≥ 60 ^c,d^	125 (53.0)	2025 (34.5)	2.1 (1.6–2.8)	<0.001	122 (52.8)	1465 (43.2)	1.5 (1.1–1.9)	0.004
MetS	197 (78.8)	2654 (41.9)	5.2 (3.8–7.0)	<0.001	193 (78.8)	2209 (60.2)	2.5 (1.8–3.4)	<0.001
ASCVD	84 (33.6)	531 (8.4)	5.5 (4.2–7.3)	<0.001	84 (34.3)	489 (13.3)	3.4 (2.6–4.5)	<0.001
CHD	43 (17.2)	278 (4.4)	4.5 (3.2–6.4)	<0.001	43 (17.6)	259 (7.1)	2.8 (2.0–4.0)	<0.001
Stroke	47 (18.8)	203 (3.2)	7.0 (4.9–9.9)	<0.001	47 (19.2)	186 (5.1)	4.4 (3.1–6.3)	<0.001
PAD	22 (8.8)	128 (2.0)	4.7 (2.9–7.5)	<0.001	22 (9.0)	119 (3.2)	2.9 (1.8–4.7)	<0.001
Erectile dysfunction ^e,f^	68 (62.4)	436 (15.6)	9.0 (6.0–13.4)	<0.001	68 (64.8)	408 (24.7)	6.6 (3.7–8.5)	<0.001
Heart failure	84 (33.6)	100 (1.6)	31.6 (22.7–44.8)	<0.001	84 (34.3)	97 (2.6)	19.2 (13.8–26.8)	<0.001
Albuminuria	61 (24.4)	333 (5.3)	5.8 (4.3–7.9)	<0.001	61 (24.9)	272 (7.4)	4.1 (3.0–5.7)	<0.001
Low eGFR	87 (34.8)	437 (6.9)	7.2 (5.5–9.5)	<0.001	87 (35.5)	424 (11.6)	4.2 (3.2–5.6)	<0.001
CKD	113 (45.2)	643 (10.1)	7.3 (5.6–9.5)	<0.001	113 (46.1)	575 (15.7)	4.6 (3.5–6.0)	<0.001
BPLT	215 (86.0)	2124 (33.5)	12.2 (8.5–17.5)	<0.001	211 (86.1)	1912 (52.1)	5.7 (3.9–8.2)	<0.001
LLT	128 (51.2)	1724 (27.2)	2.8 (2.2–3.6)	<0.001	127 (51.8)	1566 (42.7)	1.4 (1.1–1.9)	0.005
GLT	29 (11.6)	778 (12.3)	0.9 (0.6–1.4)	0.748	68 (27.8)	696 (19.0)	1.6 (1.2–−2.2)	<0.001
ULT ^a,b^	7 (2.9)	129 (2.1)	1.4 (0.6–3.0)	0.398	7 (2.9)	112 (3.1)	0.9 (0.4–2.0)	0.883
Low VR	1 (0.4)	2144 (33.8)	0.01 (0.00–0.06)	<0.001	0 (0.0)	152 (4.1)	NE	NE
Moderate VR	18 (7.2)	1361 (21.5)	0.3 (0.2–0.5)	<0.001	16 (6.5)	1041 (28.4)	0.2 (0.1–0.3)	<0.001
High VR	28 (11.2)	995 (15.7)	0.7 (0.5–1.0)	0.054	27 (11.0)	753 (20.5)	0.5 (0.3–0.7)	<0.001
Very high VR	203 (81.2)	1838 (29.0)	10.6 (7.7–14.6)	<0.001	202 (82.5)	1721 (46.9)	5.3 (3.8–7.4)	<0.001

No. (%): number of cases (percentage); OR: odds ratio; CI: confidence interval; *p*: *p*-value of the difference in percentage, ^a^ N (≥18 yr): 245 with AF; 6244 without AF, ^b^ N (≥50 yr): 240 with AF; 3619 without AF, ^c^ N (≥18 yr): 236 with AF; 5872 without AF, ^d^ N (≥50 yr): 231 with AF; 3393 without AF, ^e^ N (men ≥ 18 yr): 109 with AF; 2795 without AF, ^f^ N (men ≥ 50 yr): 105 with AF; 1651 without AF. AF: atrial fibrillation; ASCVD: atherosclerotic cardiovascular disease; BPLT: blood pressure-lowering drug therapy; CHD: coronary heart disease; CKD: chronic kidney disease; CUN-BAE: according to its acronym in Spanish, Clínica Universitaria de Navarra—Body Adiposity Estimator; eGFR: estimated glomerular filtration rate; GLT: glycaemic-lowering drug therapy; HDL-C: high-density lipoprotein cholesterol; LLT: lipid-lowering drug therapy; MetS: metabolic syndrome; NE: not estimable; PAD: peripheral arterial disease; ULT: urate-lowering drug therapy; VR: vascular risk; WHtR: waist-to-height ratio. The definitions of the cardiovascular–kidney–metabolic factors, comorbidities, or clinical conditions are shown in Table S1 (Supplementary Materials).

**Table 3:** Multivariate analysis of cardiovascular–kidney–metabolic factors on atrial fibrillation.

Population ≥ 18 yr	Wald	β ^a^	OR Exp (β) ^b^	*p* ^c^	Population ≥ 50 yr	Wald	β ^a^	OR Exp (β) ^b^	*p* ^c^
Heart failure	175.4	2.48 (0.19)	11.97 (8.29–17.28)	<0.001	Heart failure	164.7	2.37 (0.19)	10.74 (7.48–15.44)	<0.001
Hypertension	44.2	1.19 (0.18)	3.27 (2.31–4.64)	<0.001	CKD	30.3	0.86 (0.16)	2.37 (1.74–3.22)	<0.001
CKD	37.6	0.97 (0.16)	2.63 (1.93–3.58)	<0.001	Stroke	16.1	0.85 (0.21)	2.34 (1.55–3.56)	<0.001
Stroke	17.5	0.90 (0.22)	2.47 (1.62–3.77)	<0.001	Hypertension	9.3	0.55 (0.18)	1.74 (1.22–2.48)	0.002
Low HDL-C	14.1	0.55 (0.15)	1.73 (1.30–2.30)	<0.001	Low HDL-C	13.0	0.53 (0.15)	1.70 (1.27–2.27)	<0.001
Prediabetes	6.0	0.38 (0.15)	1.46 (1.08–2.00)	0.014					

^a^ β coefficient (± deviation). ^b^ Odds ratio Exp (β) (95% confidence interval). ^c^ *p*: *p*-value of Wald test with one degree of freedom. CKD: chronic kidney disease; HDL-C: high-density lipoprotein cholesterol. The definitions of the cardiovascular–kidney–metabolic factors, comorbidities, or clinical conditions are shown in Table S1 (Supplementary Materials).

## Data Availability

No new data were created or analyzed in this study. Data sharing is not applicable to this article.

## Supplementary Materials

The following supporting information can be downloaded at https://www.mdpi.com/article/10.3390/medicina60081309/s1. Extended methods explanation; Table S1: Definitions and criteria of clinical variables and conditions; Table S2: Prevalence rates of atrial fibrillation according to age-groups. References [1,2,7,13,14,46,47,48,49,50,51,52,53,54,55,56,57] are cited in Supplementary Materials.
