# Supplementary material for: Atrial Fibrillation Prevalence Rates and Its Association with Cardiovascular–Kidney–Metabolic Factors: SIMETAP-AF Study
Source: Medicina (Kaunas). 2024 Aug 13;60(8):1309. doi: 10.3390/medicina60081309 (PMC11356659; doi:10.3390/medicina60081309)
Supplement: Supplementary file 1 [file medicina-60-01309-s001.zip › medicina-3125185-supplementary.pdf]

Atrial fibrillation prevalence rates and its association with cardiovascular-kidney-metabolic factors. SIMETAP-AF Study.

SUPPLEMENTARY MATERIAL

Table S1. Definitions and criteria of clinical variables and conditions

Table S2. Prevalence rates of atrial fibrillation according to age-groups

Methods

1.1 *Sampling, Recruitment and Data Collection.* All people aged 18 or over assigned to the researchers were selected by simple random sampling from Excel's *randbetween* function. Inclusion criteria: The order indicated by the lists of random numbers was applied to include the study subjects until reaching the sample size necessary to evaluate the objectives of the study. Patients with terminal illnesses or cognitive impairment, institutionalised people, dementia, schizophrenia or moderate or severe psychosis, residents of nursing homes, pregnant women, and people who are participating in other clinical studies were excluded per-protocol. Finally, 6,588 people aged 18 years or over were recruited with informed consent and with the necessary clinical and laboratory data to be evaluated (population response rate 62.9%). The most recent data from the previous year were collected from January to December 2015. All information assessed in this study were collected from the primary care electronic health records under real-world data setting.

1.2 *Statistical Analysis.* Qualitative variables were analysed using percentage of each category, Chi-square test and odds ratios (OR), with a 95% confidence interval (CI). Shapiro–Wilk test was used to check the data fitting to normal distribution for quantitative variables. If the variables showed normal distribution, they were analysed using the arithmetic mean, standard deviation (SD) and the Student's t-test or analysis of variance. Median and interquartile range (IQR) of age were determined. Prevalence rates were determined in populations ≥ 18, ≥ 50, ≥ 60, and ≥ 70 years of age. The age- and sex-adjusted prevalence rates were calculated by direct method, according to data on inhabitants of Spain in January 2015 reported by the National Institute of Statistics ([1]. Bivariate and multivariate analysis were performed for both populations ≥18 years and ≥50 years. To assess the individual effect of comorbidities and clinical conditions on the dependent variable (AF), multivariate logistic regression analysis was performed using the backward stepwise method, initially introducing into the model all the variables that showed association in the bivariate analysis up to a p-value < 0.10, except for complex variables such as metabolic syndrome (MetS) [2] or CUN-BAE (according to its acronym in Spanish, *Clínica Universitaria de Navarra* - Body Adiposity Estimator) obesity/adiposity [3], that include parameters that were individually assessed in the analysis, and for erectile dysfunction, because it affects only men. Subsequently, the variable that contributed least to the fit of the analysis was eliminated at each step. All tests were considered statistically significant if the two-tailed p-value was < 0.05. Statistical analysis was performed using the SPSS statistical package.

1. Instituto Nacional de Estadística. INEbase. Demografía y población. Cifras de población y Censos demográficos. Cifras de población. (Accessed on March 20, 2024). Available online: http://www.ine.es/dynt3/inebase/es/index.htm?padre=1894&capsel=1895.
2. Alberti KGMM, Eckel RH, Grundy SM, Zimmet PZ, Cleeman JI, Donato KA, et al. Harmonizing the metabolic syndrome: A joint interim statement of the International Diabetes Federation task force on Epidemiology and Prevention; National Heart, Lung, and Blood Institute; American Heart Association; World Heart Federation; International Atherosclerosis Society; and International Association for the Study of Obesity. Circulation. 2009; 120:1640–5.
3. Gómez-Ambrosi J, Silva C, Catalán V, Rodríguez A, Galofré JC, Escalada J, et al. Clinical usefulness of a new equation for estimating body fat. Diabetes Care. 2012; 35:383–8.

Table S1

Definitions and criteria of clinical variables and conditions

**Morbidities, variables or clinical conditions**	**Concepts and criteria that define the variables**
Current smoking	Any amount of tobacco use in the previous year.
Alcoholism	> 21 standard drink units (SDU) of alcohol consumption per week (male), > 14 SDU per week (female).<br>1 SDU is equivalent to 10 g of alcohol.
Physical inactivity (1)	Moderate-intensity physical activity (e.g., brisk walking) < 150 minutes a week, or vigorous-intensity physical activity (e.g., jogging) < 75 minutes a week (or less of equivalent combination of both), or muscle strengthening exercises < 2 days a week.
Overweight (2)	Body mass index (BMI) 25.0–29.9 kg/m^2 (International Classification of Diseases, 10th Revision, Clinical Modification [ICD-10-CM]: E66.3; International Classification of Primary Care, 2nd edition [ICPC-2]: T83)*
Obesity (2)	BMI ≥ 30 kg/m^2 (ICD-10-CM: E66.9; ICPC-2: T82)*
Abdominal or central obesity (3)	Increased waist circumference (≥102 cm [male]; ≥88 cm [female]) determined with the subject standing using a flexible tape measure adjusted without compressing the skin, at the end of a normal expiration, locating the upper edge of the iliac crests and above that point surrounding the waist parallel to the floor.
Adiposity CUN-BAE (4)	Adiposity or body fat index CUN-BAE (according to its acronym in Spanish, *Clínica Universitaria de Navarra* - Body Adiposity Estimator):<br>• Male: −44.988 + (0.503 x age) + (3.172 x BMI) − (0.026 x BMI^2) − (0.02 x BMI x age) + (0.00021 x BMI^2 x age)<br>• Female: −44.988 + (0.503 x age) + 10.689 + (3.172 x BMI) − (0.026 x BMI^2) + (0.181 x BMI) − (0.02 x BMI x age) − (0.005 x BMI^2) + (0.00021 x BMI^2 x age)
Excess adiposity CUN-BAE (4)	Adiposity or body fat index CUN-BAE > 25% for men or > 35% for female
High waist-to-height ratio (WHtR) (5)	Waist circumference/height ≥ 0.55
Arterial hypertension (HTN) (6)	Systolic blood pressure (SBP) ≥ 140 mmHg and/or diastolic blood pressure (DBP) ≥ 90 mmHg, using the average of two or more readings obtained on two or more occasions, or being on antihypertensive treatment (ICD-10-CM: I10, I15; ICPC-2: K86, K87)*
Pulse pressure	SBP – DBP (mmHg)

Diabetes mellitus (DM) (7)	According to the American Diabetes Association (ADA) criteria: fasting plasma glucose (FPG) ≥ 126 mg/dL (7.0 mmol/L) or glycated haemoglobin A_{1c} (HbA_{1c}) ≥ 6.5 % (in International Federation of Clinical Chemistry and Laboratory Medicine [IFCC] units) (≥ 48 mmol/mol) or plasma glucose ≥ 200 mg/dL (11.1 mmol/L) at any time or with oral glucose tolerance test (ICD-10-CM: E10, E11; ICPC-2: T89, T90)*<br>To convert from mg/dL to mmol/L, multiply by 0.05556<br>To convert from % (DCCT) to mmol/mol (IFCC), subtract 2.15 and multiply by 10.929
Prediabetes (7)	According to the ADA criteria: FPG between 100 and 125 mg/dL or HbA_{1c} between 5.7% and 6.4% (ICD-10-CM: R73.09; ICPC-2: A91)*<br>To convert from mg/dL to mmol/L, multiply by 0.05556<br>To convert from % (DCCT) to mmol/mol (IFCC), subtract 2.15 and multiply by 10.929
Estimated average glucose (eAG)	28.7 x HbA_{1c} – 46.7 (mg/dL)<br>To convert from mg/dL to mmol/L, multiply by 0.05556
Hypercholesterolaemia	Fasting plasma total cholesterol (TC) concentration ≥ 200 mg/dL (≥ 5.17 mmol/L) (ICD-10-CM: E78; ICPC-2: T93)*<br>To convert from mg/dL to mmol/L, multiply by 0.02586
Hypertriglyceridaemia (HTG)	Fasting plasma triglyceride (TG) concentration ≥ 150 mg/dL (≥ 1.69 mmol/L) (ICD-10-CM: E78; ICPC-2: T93)*<br>To convert from mg/dL to mmol/L, multiply by 0.01129
Low high-density lipoprotein cholesterol (HDL-C)	HDL-C < 40 mg/dL (< 1.03 mmol/L) (male)<br>HDL-C < 50 mg/dL (< 1.29 mmol/L) (female)<br>To convert from mg/dL to mmol/L, multiply by 0.02586
Atherogenic dyslipidaemia	HTG and low HDL-C
Non-high-density lipoprotein cholesterol (Non-HDL-C)	TC – HDL-C
Low-density lipoprotein cholesterol (LDL-C)	TC – HDL-C – (TG/5) mg/dL (not valid for patients with TG > 400 mg/dL)<br>TC – HDL-C – (TG/2.2) mmol/L (not valid for patients with TG > 4.51 mmol/L)<br>TC, HDL-C: To convert from mg/dL to mmol/L, multiply by 0.02586<br>TG: To convert from mg/dL to mmol/L, multiply by 0.01129
Residual cholesterol (RC)	Very low-density lipoproteins cholesterol (VLDL-C) and remnants.<br>RC= TC – HDL-C – LDL-C
Triglyceride-glucose (TyG) index	Ln (TG x FPG/2)

Atherogenic index of plasma (AIP)	log TG/HDL
Metabolic syndrome (MetS) (3)	According to Harmonized Consensus of International Diabetes Federation task force on Epidemiology and Prevention, National Heart, Lung, and Blood Institute, American Heart Association, World Heart Federation, International Atherosclerosis Society, and International Association for the Study of Obesity (3).<br>At least, three of following factors for the European population:<br>• Increased waist circumference (≥ 102 cm [male]; ≥ 88 cm [female])<br>• FPG ≥ 100 mg/dL (≥ 5.6 mmol/L)<br>• TG ≥ 150 mg/dL (≥ 1.7 mmol/L)<br>• HDL-C < 40 mg/dL (< 1.03 mmol/L) (males); < 50 mg/dL (< 1.29 mmol/L) (females)<br>• SBP ≥ 130 mmHg or DBP ≥ 85 mmHg or antihypertensive treatment
Fatty liver index (FLI) (8)	$FLI = (e^{0.953*loge\ (TG) + 0.139 \times BMI + 0.718 \times loge\ (GGT) + 0.053 \times waist\ circumference - 15.745}) / (1 + e^{0.953*loge\ (TG) + 0.139 \times BMI + 0.718 \times loge\ (GGT) + 0.053 \times waist\ circumference - 15.745}) \times 100$<br>$log_e$ = natural logarithm; GGT = gamma-glutamyl-transferase<br>A value FLI between 0 and 30 can be used to rule out steatotic liver disease (SLD) (sensitivity: 87%; negative likelihood ratio: 0.2) and a value FLI between 60 and 100 to rule in SLD (specificity 86%; positive likelihood ratio: 4.3).
Hyperuricaemia (HU) (9)	Serum uric acid (SUA) levels ≥ 7.0 mg/dL (416 µmol/L) for both adult males and females, or urate-lowering therapy (ULT) (ICD-10-CM: E79; ICPC-2: T92)*<br>To convert from mg/dL to mmol/L, multiply by 0.05948
Coronary heart disease (CHD)	Ischemic heart disease, acute myocardial infarction, acute coronary syndrome, coronary revascularization (ICD-10-CM: I20-I25; ICPC-2: K74, K75, K76)*
Cerebrovascular disease (stroke)	Cerebral ischemia, intracranial haemorrhage, transient ischemic attack (ICD-10-CM: I60-I66, I66, I67; ICPC-2: K89, K90K K91)*
Peripheral arterial disease (PAD)	Intermittent claudication, ankle-brachial index ≤ 0.9 (ICD-10-CM: I70.2, I73.9; ICPC-2: K92)*
Atherosclerotic cardiovascular disease (ASCVD)	ASCVD include CHD, stroke, or PAD (ICD-10-CM: I70)*
Heart failure (HF) (10)	Record of HF diagnosis (ICD-10-CM: I50; ICPC-2: K77)* in the patient's medical record, without differentiating by phenotype based on measurement of left ventricular ejection fraction or based on severity of symptoms and physical activity.
Hospitalization for HF (hHF) (10)	Admission for ≥ 24 h with a primary diagnosis of HF, with ≥1 symptom and ≥ 2 physical examination, laboratory, or invasive findings of HF, and receives a HF-specific treatment.

Atrial fibrillation (AF) (11,12)	Record of AF diagnosis (ICD-10-CM: I48; ICPC-2: K78)* in the patient's medical record, without differentiating by phenotypes based on paroxysmal, persistent, long-standing persistent, or permanent AF or atrial flutter.
Estimated glomerular filtration rate (eGFR) (13)	According to Chronic Kidney Disease Epidemiology Collaboration (CKD-EPI) equations:<br>Women with creatinine ≤ 0.7 mg/dL= 144 x $(\text{creatinine})^{-0.329}$ x $(0.993)^{\text{age}}$ mL/min/1.73 m^2 of the body surface<br>Women with creatinine > 0.7 mg/dL= 144 x $(\text{creatinine})^{-1.209}$ x $(0.993)^{\text{age}}$ mL/min/1.73 m^2 of the body surface<br>Men with creatinine ≤ 0,9 mg/dL= 141 x $(\text{creatinine})^{-0,411}$ x $(0,993)^{\text{age}}$ mL/min/1.73 m^2 of the body surface<br>Men with creatinine > 0,9 mg/dL= 141 x $(\text{creatinine})^{-1,209}$ x $(0,993)^{\text{age}}$ mL/min/1.73 m^2 of the body surface
Low eGFR (14)	eGFR < 60 mL/min/1.73 m^2 according to CKD-EPI [12]<br>Not includes:<br>• Stage G1: ≥ 90 mL/min/1.73 m^2<br>• Stage G2: 60 to 89 mL/min/1.73 m^2<br>Includes:<br>• Stage G3a: 45 to 59 mL/min/1.73 m^2<br>• Stage G3b: 30 to 44 mL/min/1.73 m^2<br>• Stage G4: 15 to 29 mL/min/1.73 m^2<br>• Stage G5: < 15 mL/min/1.73 m^2
Albuminuria (14)	Urine albumin-creatinine ratio (ACR) ≥ 30 mg/g (including proteinuria [ACR > 300 mg/g] (ICD-10-CM: R80; ICPC-2: U98)*<br>Not includes:<br>• Stage A1: < 30 mg/g<br>Includes:<br>• Stage A2: 30 mg/g to 300 mg/g<br>• Stage A3: > 300 mg/g<br>To convert from mg/g to mg/mmol, multiply by 0.01131
Chronic kidney disease (CKD) (14)	Low eGFR and/or albuminuria (ICD-10-CM: N18; ICPC-2: U99)*
Cardiovascular-kidney-metabolic (CKM) syndrome (15)	Systemic disorder attributable to pathophysiological interactions among metabolic risk factors, CKD, and CVD, that includes both individuals at risk for CVD, CKD, and those with existing clinical CVD.<br>• Stage 0: BMI <25 kg/m^2, normal abdominal circumference (<88 in women and <102 cm in men) without criteria for the other stages.<br>• Stage 1: adiposity excess (CUN-BAE obesity), overweight, obesity, abdominal obesity, or prediabetes.<br>• Stage 2: HTN, metabolic risk factors (HTG, DM, MetS), moderate or high risk CKD.<br>• Stage 3: subclinical target organ damage, risk equivalents (high vascular risk [VR] or very high-risk CKD) among individuals with stages 1 or 2.

	• Stage 4: clinical CVD including CHD, stroke, PAD, HF, and AF among individuals with stages 1 or 2 (stage 4a: without CKD; stage 4b: with CKD).<br>• Stages 3 or 4 are defined as advanced stages of CKM syndrome because they identify individuals at high risk for CVD.
Vascular risk (VR) categories (previously called cardiovascular risk) (16)	Low, moderate, high and very high VR was estimated for patients from low-risk European countries according to 2021 ESC Guidelines on cardiovascular disease prevention in clinical practice.

* National Center for Health Statistics (NCHS). International Classification of Diseases, Tenth Revision, Clinical Modification (ICD-10-CM). [Accessed June 10, 2024]. Available from: https://www.cdc.gov/nchs/icd/icd-10-cm.htm#print.

* World Health Organization. (2009). International Classification of Primary Care, 2nd edition - ICPC-2. [Accessed June 10, 2024]. Available from: https://www.who.int/standards/classifications/other-classifications/international-classification-of-primary-care.

References

1. World Health Organization. WHO guidelines on physical activity and sedentary behaviour. World Health Organization. **2020**. [Accessed June 10, 2024]. Available from: https://iris.who.int/handle/10665/336656.
2. WHO Consultation on Obesity (1999: Geneva, Switzerland) & World Health Organization). Obesity: preventing and managing the global epidemic: Report of a WHO consultation. WHO technical report series; 894. **2000**. [Accessed June 10, 2024]. Available from: https://apps.who.int/iris/handle/10665/42330.
3. Alberti KGMM, Eckel RH, Grundy SM, Zimmet PZ, Cleeman JI, Donato KA, et al. Harmonizing the metabolic syndrome: A joint interim statement of the International Diabetes Federation task force on Epidemiology and Prevention; National Heart, Lung, and Blood Institute; American Heart Association; World Heart Federation; International Atherosclerosis Society; and International Association for the Study of Obesity. Circulation. **2009**; 120:1640–5, https://doi.org/10.1161/CIRCULATIONAHA.109.192644.
4. Gómez-Ambrosi J, Silva C, Catalán V, Rodríguez A, Galofré JC, Escalada J, et al. Clinical usefulness of a new equation for estimating body fat. Diabetes Care. **2012**; 35:383–8, https://doi.org//10.2337/dc11-1334.
5. Romero-Saldaña M, Fuentes-Jiménez FJ, Vaquero-Abellán M, Álvarez-Fernández C, Aguilera-López MD, Molina-Recio G. Predictive capacity and cutoff value of waist-to-height ratio in the incidence of metabolic syndrome. Clin Nurs Res. **2019**; 28:676–91, https://doi.org/10.1177/1054773817740533.
6. Mancia G, Kreutz R, Brunström M, Burnier M. Grassi G, Januszewicz A, et al. 2023 ESH Guidelines for the management of arterial hypertension. The Task Force for the management of arterial hypertension of the European Society of Hypertension. Endorsed by the European Renal Association (ERA) and the International Society of Hypertension (ISH). J Hypertension. J Hypertens. **2023**; 41(12):1874-2071. https://doi.org/10.1097/HJH.0000000000003480
7. American Diabetes Association Professional Practice Committee. 2. Diagnosis and classification of diabetes: Standards of Care in Diabetes—2024. Diabetes Care. **2024**; 47(Suppl. 1):S20–S42, https://doi.org/10.2337/dc24-S002

8. Bedogni G, Bellentani S, Miglioli L, Masutti F, Passalacqua M, Castiglione A, et al. The Fatty Liver Index: a simple and accurate predictor of hepatic steatosis in the general population. BMC Gastroenterol. **2006**; 6:33. https://doi.org/10.1186/1471-230X-6-33.
9. Khanna D, Fitzgerald JD, Khanna PP, Bae S, Singh MK, Neogi T, et al. 2012 American College of Rheumatology Guidelines for Management of Gout Part I: systematic nonpharmacologic and pharmacologic therapeutic approaches to hyperuricemia. Arthritis Care Res. **2012**; 64,1431–46, https://doi.org/10.1002/acr.21772.
10. Abraham WT, Psotka MA, Fiuzat M, Filippatos G, Lindenfeld J, Mehran R, et al. Standardized definitions for evaluation of heart failure therapies: Scientific Expert Panel from the Heart Failure Collaboratory and Academic Research Consortium. JACC Heart Fail. **2020**; 8(12):961-972. https://doi.org/10.1016/j.jchf.2020.10.002.
11. Hindricks G, Potpara T, Dagres N, Arbelo E, Bax JJ, Blomström-Lundqvist C, et al.; ESC Scientific Document Group. 2020 ESC Guidelines for the diagnosis and management of atrial fibrillation developed in collaboration with the European Association for Cardio-Thoracic Surgery (EACTS): The Task Force for the diagnosis and management of atrial fibrillation of the European Society of Cardiology (ESC). Developed with the special contribution of the European Heart Rhythm Association (EHRA) of the ESC. Eur. Heart. J. **2021**; 42:373–498. https://doi.org/10.1093/eurheartj/ehaa612.
12. Joglar JA, Chung MK, Armbruster AL, Benjamin EJ, Chyou JY, Cronin EM, et al. 2023 ACC/AHA/ACCP/HRS Guideline for the diagnosis and management of atrial fibrillation: A report of the American College of Cardiology/American Heart Association Joint Committee on Clinical Practice Guidelines. Circulation. **2024**; 149(1):e1-e156. https://doi.org/10.1161/CIR.0000000000001193.
13. Levey AS, Stevens LA, Schmid CH, Zhang YL, Castro AF 3rd, Feldman HI, et al., CKD-EPI (Chronic Kidney Disease Epidemiology Collaboration). A new equation to estimate glomerular filtration rate. Ann Intern Med. **2009**; 150:604–12, https://doi.org/10.7326/0003-4819-150-9-200905050-00006.
14. Kidney Disease: Improving Global Outcomes (KDIGO) CKD Work Group. KDIGO 2024 Clinical Practice Guideline for the Evaluation and Management of Chronic Kidney Disease. Kidney Int. **2024**; 105(4S):S117-S314. https://doi.org/10.1016/j.kint.2023.10.018.
15. Ndumele CE, Rangaswami J, Chow SL, Neeland IJ, Tuttle KR, Khan SS, et al.; American Heart Association. Cardiovascular-kidney-metabolic health: A Presidential Advisory from the American Heart Association. Circulation. **2023**; 148(20):1606-1635. https://doi.org/10.1161/CIR.0000000000001184.
16. Visseren FLJ, Mach F, Smulders YM, Carballo D, Koskinas KC, Bäck M, et al; ESC Scientific Document Group. 2021 ESC Guidelines on cardiovascular disease prevention in clinical practice. Developed by the Task Force for cardiovascular disease prevention in clinical practice with representatives of the European Society of Cardiology and 12 medical societies. With the special contribution of the European Association of Preventive Cardiology (EAPC). Eur Heart J. **2021**; 42:3227–337. https://doi.org/10.1093/eurheartj/ehab484.

Table S2

Prevalence rates of atrial fibrillation according to age-groups

Age-group (yr)	Crude prevalence rates				Adjusted prevalence rates		
	Overall % (95% CI)	Male % (95% CI)	Female % (95% CI)	*p*	Overall (%)	Male (%)	Female (%)
**≥ 18**	3.79 (3.33–4.26)	3.75 (3.06–4.44)	3.83 (3.21–4.45)	0.866	2.89	2.68	3.11
**< 50**	0.19 (0.02–0.35)	0.35 (0.01–0.69)	0.07 (-0.06 – 0.19)	0.093	0.09	0.19	0.03
**≥ 50**	6.26 (5.50–7.02)	5.98 (4.87–7.09)	6.49 (5.45–7.53)	0.513	6.09	5.72	6.42
**≥ 60**	8.34 (7.29–9.39)	7.71 (6.18–9.24)	8.64 (7.40–10.27)	0.296	8.54	7.76	9.14
**≥ 70**	12.80 (11.09–14.51)	13.00 (10.31–15.69)	12.66 (10.44–14.88)	0.848	12.92	12.88	12.95

yr: years; CI: confidence interval; *p*: *p*-value of difference in percentages
